# Ceramides in Adipose Tissue

**DOI:** 10.3389/fendo.2020.00407

**Published:** 2020-06-19

**Authors:** Ying Li, Chad Lamar Talbot, Bhagirath Chaurasia

**Affiliations:** ^1^Department of Nutrition and Integrative Physiology, The Diabetes and Metabolism Research Center, University of Utah, Salt Lake City, UT, United States; ^2^Division of Endocrinology, Department of Internal Medicine, Fraternal Order of Eagles Diabetes Research Center, Carver College of Medicine, University of Iowa, Iowa City, IA, United States

**Keywords:** metabolism, adipocytes, diabetes, insulin, ceramides

## Abstract

Adipose tissue is a key nutrient-sensing depot that regulates excess energy storage and consumption. Adipocytes, the key components of the adipose tissue, have unique ability to store excess energy in the form of triglycerides, sense systemic energy demands, and secrete factors (lipids, peptides, cytokines, and adipokines) to regulate other metabolic tissues. The presence of various types of adipocytes (white, brown, and beige) characterized by the number/size of lipid droplets, mitochondrial density, and thermogenic capacity, further highlights how intricate is the communication of these cell-types with other metabolic tissues to sense nutrients. In obesity the inherent capacity of adipose tissue to store and sense nutrients is compromised, causing spillover of the intermediate lipid metabolites into circulation and resulting in their ectopic deposition in tissues not suitable for lipid storage, a phenomenon known as lipotoxicity. This results in a spectrum of cellular dysfunction, that underlies various metabolic diseases. Of the numerous lipid classes implicated in eliciting lipotoxicity, sphingolipid: ceramides are among the most deleterious as they modulate signaling pathways involved in regulating glucose metabolism, triglyceride synthesis, apoptosis, and fibrosis. Notably, recent experimental studies have strongly implicated ceramides in the development of numerous metabolic diseases such as insulin resistance, diabetes, cardiomyopathy, hepatic-steatosis, and atherosclerosis. Herein we discuss and summarizes recent findings that implicate ceramides as a key contributor to adipocyte dysfunction underlying metabolic diseases and how depletion of ceramides can be exploited to improve metabolic health.

## Introduction

Metabolic diseases represent a significant health burden that impacts millions of households worldwide. According to the World Health Organization (WHO) global report, ~422 million adults were living with diabetes in 2014 (1), and that cardiovascular diseases account for 31% mortality worldwide (2). The epidemic of obesity is one of the major causes of these metabolic disorders. Accumulation of neutral lipids, such as triglycerides, in key insulin target tissues, has been postulated to inhibit metabolic functions, however, they are less likely to be deleterious. On the other hand, there is growing evidence for the involvement of other lipid metabolites in inducing this metabolic outburst (3–5). Notably, recent studies suggest that the accumulation of sphingolipids, namely ceramides and it's metabolites, play essential roles in the development of insulin resistance in tissues such as skeletal muscle, liver and, adipose tissue in obese rodents, and humans (6–20). In mice, blocking ceramide production improves insulin sensitivity, prevents β-cell failure, resolves hepatic steatosis, hypertriglyceridemia, and prevents atherosclerosis, and heart failure (6–22). Enhancing ceramide degradation also endows these metabolic benefits, and adiponectin exerts their antidiabetic, cardioprotective, and insulin-sensitizing actions through activating its receptors, which are ligand-activated ceramidases (23). In humans, ceramides predict the occurrence of major adverse cardiac events, such that the numerous clinics have started to offer serum ceramide tests as prognostic measures of cardiovascular risk (24, 25).

In this review, we intend to provide a perspective on ceramides and ceramide metabolites in the maintenance of adipose tissue homeostasis and how adipose tissue ceramides contribute to the development of metabolic diseases.

## Clinical Association Of Adipose Ceramide Content With Obesity and Insulin Resistance

As it is apparent in this series, numerous studies have shown that manipulation of ceramide synthesis or degradation pathways in rodents through pharmacologic and genetic interventions have profound effects on insulin sensitivity (6, 12, 26, 27). Despite the lack of these interventions in humans, clinical studies highlight a strong association between serum/plasma ceramides and adverse outcomes in metabolic and cardiovascular diseases (28–33). In adipose tissue, ceramide content has been associated with the development of insulin resistance in numerous human studies consisting of small cohorts. In one of these studies, Yki-Jävinen et al. profiled adipose tissue from 20 individuals of Finish descent and demonstrated that adipose tissue ceramides are elevated in individuals with insulin resistance independent of obesity (34). Consistent with this observation, we found that in a small cohort of individuals with Asian descent (18 individuals) various ceramide species are elevated in the adipose tissues of subjects with type 2 diabetes independent of obesity (35). Furthermore, Brüning et al. profiled 20 individuals of European descent and found that various ceramide species are elevated in the adipose tissues of obese individuals (20). Brüning et al. went on to further demonstrate that in a larger cohort of similar descent (439 individuals), mRNA expression of *CERS6* in adipose tissue positively correlates with Body Mass Index, body fat content, and hyperglycemia while negatively correlating with glucose infusion rate during euglycemic-hyperinsulinemic clamps (20).

## Regulation of Ceramide Synthesis in Adipose Tissue

**Figure 1:**
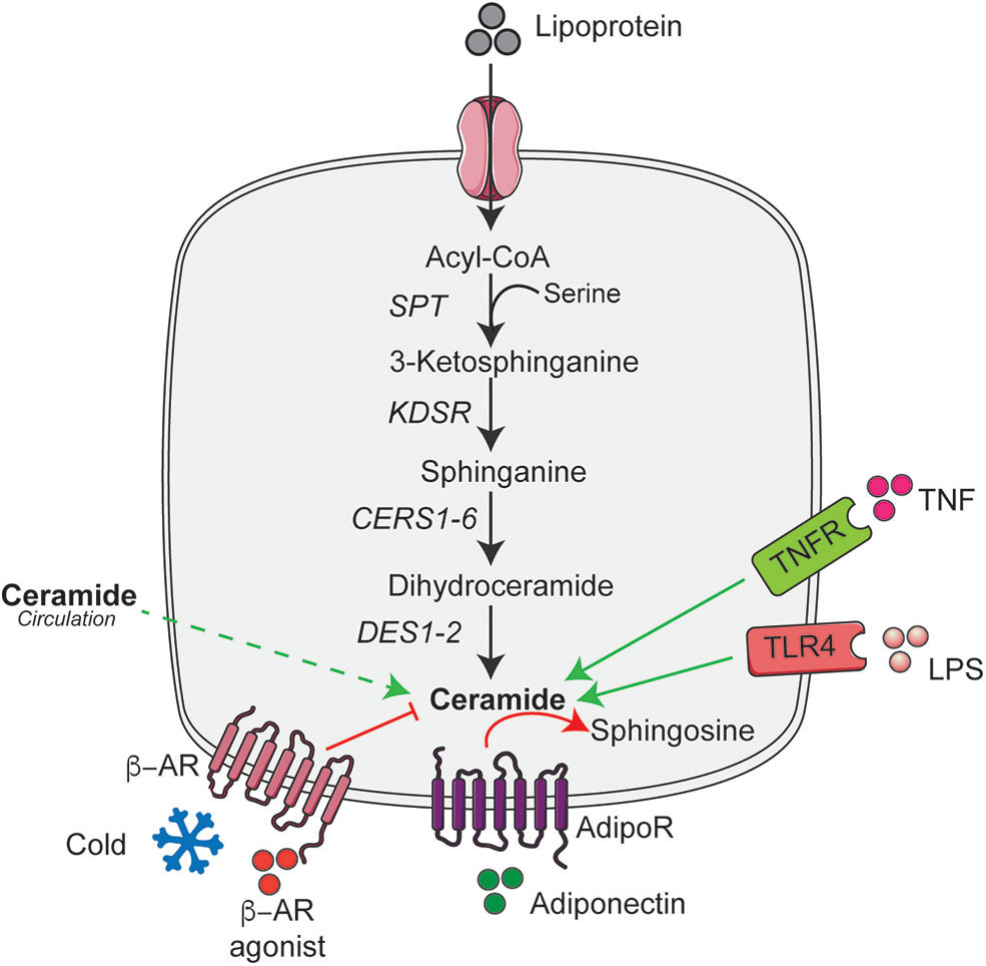
*De novo* ceramide synthesis pathway and its hormonal regulators in adipose tissue. Schematic of the *de novo* ceramide synthesis pathway and regulators of ceramide synthesis discussed in the text. Black arrow indicates the key steps in *de novo* ceramide synthesis; solid green arrow indicates the factors that stimulate ceramide synthesis; dashed green arrow indicate the putative uptake and red arrows and lines indicate factors that reduce or metabolize ceramide. AdipoR, Adiponectin receptor; ASAH1, Acid ceramidase 1; β-AR, β-adrenergic receptor; CERS1-6, Ceramide synthase 1-6; DES1-2, Dihydroceramide desaturase 1-2; KDSR, 3-ketodihydrosphingosine reductase; LPS, Lipopolysaccharide; SPT, Serine palmitoyltransferase; TLR4, Toll-like receptor-4; TNFR, Tumor necrosis factor receptor. This figure was drawn using the Servier Art.

Overnutrition is the major cause of obesity that results in the increased supply of macronutrients into the living body. They are digested, absorbed, and broken down into numerous small molecules like palmitate and serine, which are the key determinant of the elevated ceramide levels (4). Moreover, consumption of high levels of saturated fat but not unsaturated fat increases ceramide accumulation (36), while limiting the cellular serine pool reduces ceramide accumulation (37). Palmitate in addition to its role as a major substrate for ceramide synthesis also induces expression of genes involved in sphingolipid biosynthesis and metabolism (38). Although, palmitate increases ceramide accumulation in most cell types, in cultured 3T3-L1 adipocytes they do not stimulate ceramide accumulation (39). In contrast, numerous studies in rodents and humans have demonstrated an increased accumulation of ceramides in adipose tissue under conditions of nutrient excess (i.e., obesity) (20, 34, 35). Although it is not clear how nutrient excess or lipotoxic conditions increases adipose tissue ceramide content; it could potentially be due to increased trafficking of ceramides from circulation into the adipocyte. Despite this, recent studies implicate ceramides in adipose tissue act as an important secondary messenger that sense changes in nutrient status and regulates the whole-body metabolic homeostasis (35, 40). To optimally regulate nutrient homeostasis, ceramides level in the adipose tissue is tightly regulated by various systemic or intracellular signaling pathways that include a variety of hormonal factors associated with obesity and metabolic diseases independent of the dietary content (4, 6, 23, 35, 38). Here we summarize these key hormonal regulators of adipose tissue ceramide content (Figure 1).

### Ceramide and Inflammation

Adipose tissue inflammation is a hallmark of obesity characterized by the increased recruitment and activation of macrophages to adipose tissue (41). This results in increased expression and secretion of inflammatory cytokines such as tumor necrosis factor (TNF)-α, interleukins, and chemokines which increase levels of ceramides without affecting glycerolipids such as diacylglycerol (42). The excess nutrient supply in conjunction with various triggers of inflammation is a key determinant of ceramide biosynthesis and exhibits a tight association with ceramides and insulin resistance (42, 43). Moreover, saturated fatty acids that accrue in obesity induce the activation of toll-like receptors-4 (TLR4), resulting in increased inflammation and augmented mRNA expression of various enzymes involved in *de novo* ceramide biosynthesis (44, 45). Mechanistically, saturated fatty acids do not bind directly to the TLR4 receptors, but require TLR-dependent priming to induce inflammatory signaling (46). In contrast, mice lacking systemic TLR4 exhibit reduced accumulation of ceramides in response to saturated fatty acids in numerous tissues (38). These data indicate TLR4 is an essential component linking saturated fats to modulation of ceramide synthesis (47–49). However, future studies are warranted to delineate if these consequences were due to autonomous effects in adipocytes, immune cells or due to cross-talk among these cells. Interestingly in one of the earlier seminal studies, inflammatory cytokine TNF-α was also found to induce ceramide accumulation via coordinated changes in the ceramide generating (e.g., SPT) and metabolizing enzymes (e.g., sphingomyelinase) that induce hydrolysis of sphingomyelin (50–52). Mechanistically, ceramides elicit inflammation-induced insulin resistance at least in part by activation of the Nod-like receptor (Nlrp3) inflammasome that induce caspase-1 cleavage in macrophages and adipose tissue, which thereon inhibits Akt/PKB activation and results in the development of insulin resistance (53).

### Ceramides and β-adrenergic Agonists

The induction of the thermogenic program as a means to increase energy expenditure has gained notoriety in recent years given its high therapeutic potential to combat obesity. Cold exposure or β-adrenergic agonists (e.g., norepinephrine, isoproterenol, etc.), that activates β-adrenergic receptors in adipocytes, increases the thermogenic capacity of adipocytes by elevating the expression of thermogenic genes such as *Ucp1, Pgc1a*, and *Prdm16* (54). We recently found that exposing mice to cold temperatures for 5-days reduced ceramides, dihydroceramides, and sphinganine in the adipose tissue while also reduced expression of the ceramide biosynthetic genes *Sptlc2* and *CerS6* (35). We further demonstrated that systemic inhibition of ceramide biosynthesis or adipocyte-specific ablation of *Sptlc2* increased the recruitment of beige adipocytes in the adipose tissue and improved mitochondrial function (35).

In parallel with these findings, Jiang et al. demonstrated that ceramides inhibit the browning of beige adipocytes cultured *ex vivo*, suggesting that endogenous ceramides could be autonomous regulators of adipocyte function (55). Indeed, using a similar *ex vivo* assay, we found that pharmacological manipulation of endogenous ceramides content modulates beige adipocytes' function (35). More recently, using a newly developed flux assay to monitor rates of ceramide production, we found that β-adrenergic agonists rapidly and completely shut down ceramide biosynthesis in primary adipocytes by inhibiting activation of hormone sensitive lipase (HSL) (6). Collectively, the work shows that the β-adrenergic's actions on the adipocyte were cell-autonomous and driven by ceramides, but not other sphingolipids.

### Ceramides and Adiponectin

Adiponectin, the antidiabetic and cardioprotective adipokine is predominantly produced and secreted from metabolically healthy adipocytes and regulates glucose and lipid homeostasis by exerting pleiotropic effects on numerous tissues such as the liver, pancreatic β-cells, kidney, heart, bone, and immune cells (56–59). Mechanistically, these effects of adiponectin were initially thought to be mediated by AMPK, a serine/threonine kinase (59). Owing to the sequence homology between adiponectin receptors (AdipoR) and other progestin and adipoQ receptor family members (PAQR) with ceramidases, and ability of human AdipoRs to promote ceramidase activity in ceramidase deficient yeast, the Scherer group hypothesized that the adiponectin might elicit its broad spectrum of actions by deacylating ceramides (23). Herein, the Scherer group recently demonstrated that the activation of adiponectin receptors AdipoR1 and 2 via adiponectin stimulates deacylation of ceramides yielding sphingosine that can be converted to sphingosine 1-phosphate (S1P) by sphingosine kinase, an effect that is dependent on a critical residue in the predicted ceramidase motif in AdipoRs (23). In a subsequent study, the Scherer group found that overexpression of AdipoRs in the adipose tissue or liver is sufficient to reduce ceramide accumulation in these depots owing to their increased ceramidase activity (60). Conversely, acute inhibition of adiponectin in adipose tissue increased accumulation of the most abundant ceramide species (C_16:0_) in adipose tissue and reduced sphingosine content, further highlighting the presence of ceramidase activity in AdipoRs that requires adiponectin (61). Consistent with this, Tanabe et al. initially showed that crystal structures of human AdipoRs possess a hydrophobic binding pocket potentially resembling that of the ceramidases (62). Using x-ray crystallography, Vasiliauskaite-Brooks et al. recently showed that purified adiponectin receptors possess inherent ceramidase enzymatic activity (63). They further went on to solve the crystal structure of adiponectin receptors in the presence of ceramide, obtaining a final entity bound to a fatty acid product of the reaction (63). From the clinical perspective, increased ceramide accumulation in plasma and tissues is inversely correlated with adiponectin in obese and insulin resistant subjects (64, 65).

## Lowering Ceramide Accumulation Improves Adipocyte Function

**Figure 2:**
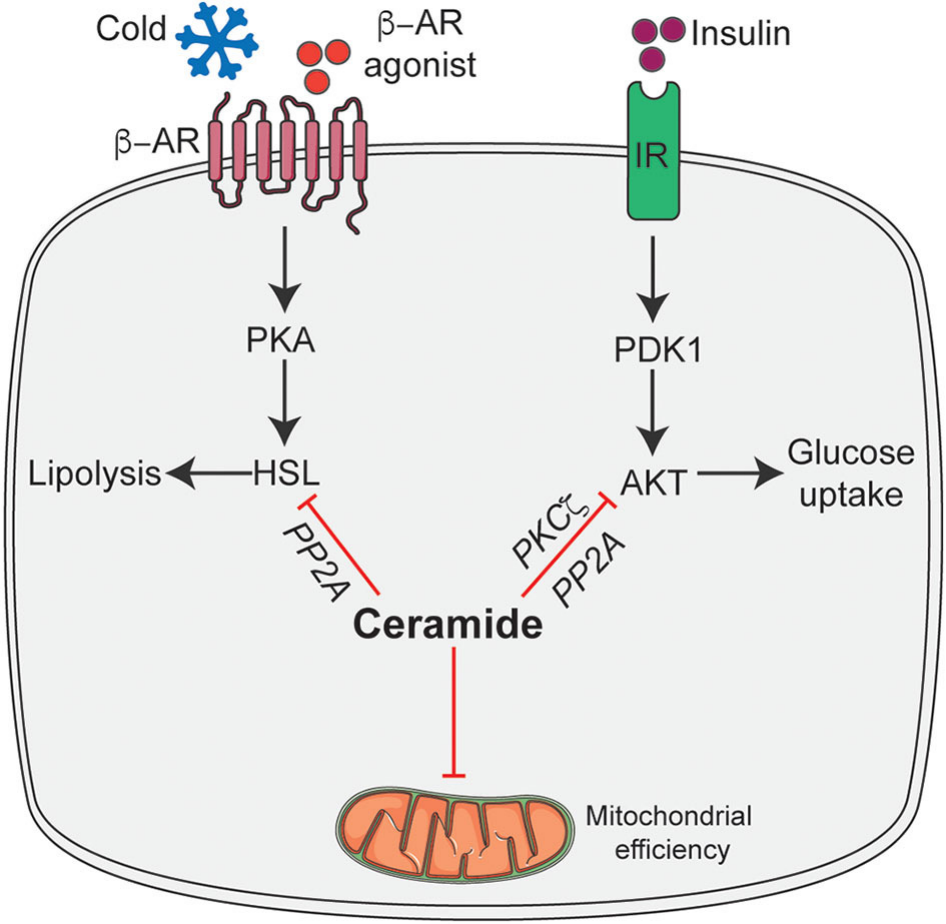
Schematic of ceramide dependent molecular mechanisms that modulate adipocyte function. Ceramides gauge adipocyte energy stores via the following mechanisms: inhibit insulin-stimulated Akt/PKB thus inhibiting glucose uptake and metabolism; inhibit HSL activation in response to β-adrenergic receptor activation thereby attenuating lipolysis and release of free fatty acid and inhibit mitochondrial efficiency. β-AR, β-adrenergic receptor; IR, Insulin receptor; HSL, Hormone sensitive lipase; Akt/PKB, Protein kinase B; PDK1, phosphoinositide dependent kinase-1; PKA, Protein kinase A; PP2A, Protein phosphatase 2A; PKCζ, protein kinase Cζ. This figure was drawn using the Servier Art.

We recently found that pharmacological inhibition of ceramide biosynthesis in obese mice, using myriocin (a selective inhibitor of SPT, the first rate-limiting enzyme in the ceramide synthetic pathway), induced profound changes in the adipose tissue. Importantly, this intervention significantly reduced adipocyte size, increased recruitment of M2 macrophages, and elevated numbers of brown/beige adipocytes in white adipose tissue, particularly in the subcutaneous depot (35). We further found that the effects observed following myriocin treatment could be recapitulated by ablation of *Sptlc2*, specifically in the adipocyte, including the improvement in insulin sensitivity and glucose tolerance, resolution of hepatic steatosis, increased recruitment of M2 macrophages, recruitment of beige adipocytes in the adipose tissue, and improvement in mitochondrial respiration (35). Interestingly, these adipocyte-specific but not macrophage-specific changes were sufficient to increase whole-body energy expenditure and resolve hepatic steatosis. The increased recruitment of M2 macrophages into adipose tissue accompanied by *Sptlc2* inhibition in the adipocyte, further highlight the cross-talk between adipocytes and macrophages to maintain adipose tissue homeostasis. However, future studies determining how adipocyte ceramides modulate M2 macrophages recruitment either via expression and secretion of an adipokine/cytokine or ceramide are warranted. In a subsequent study, we demonstrated that these newly identified ceramide effects were driven in part by PP2A dependent inhibition of hormone sensitive lipase (HSL) (6) (Figure 2). Based on these findings, we proposed that ceramides act as nutrient signals that direct the adipocyte into a diminished metabolic state, rather than an active thermogenic state.

More recently, we found that inhibition of DES1 in adipose tissue improved glucose metabolism and resolved hepatic steatosis. Interestingly, these observed improvements were independent of browning/beiging of adipose tissue (6). Using an alternative approach to selectively reduce adipose ceramides by overexpressing ASAH1 in adipose tissue, Scherer et al. found that ceramide reduction, as early as within 3-days, resolved hepatic steatosis and improved glucose tolerance (40). Again, these effects were shown to be independent of adipose tissue browning/beiging as there were no changes observed in body weight (40).

Recent papers from the two independent laboratories found that ablation of either *Sptlc1* or *2*, respectively, in adipose tissue impaired adipose differentiation and exhibited lipodystrophy (66, 67). These differentiated effects between the two studies might arise due to the difference in animal models that were used. Specifically, the aforementioned studies used an adiponectin-Cre-recombinase line from Jackson Laboratories (68) that expresses the recombinase earlier in development, compared to the adiponectin-Cre-recombinase line used in our study that is expressed late during adipocyte differentiation and was obtained from Scherer laboratory (69). We hypothesize that this accounts for the differences in phenotypes observed in these studies. In support of this, our studies in primary pre-adipocyte show that myriocin is a potent inhibitor of adipocyte differentiation whereas inhibition of ceramide synthesis in fully differentiated adipocytes promote browning/beiging (35).

### C_16_ Ceramide Is the Deleterious Species Causing Adipocyte Dysfunction

To understand which ceramide species account for the adipose tissue dysfunction, Brüning et al. profiled various ceramide species in adipose tissue isolated from rodents (20). They found that C_16_-ceramides species were highly enriched in adipose tissue, which was supported by the finding of *CerS6*, the enzyme essential for synthesizing C_16_-ceramides species, robustly elevated in various rodent models of obesity. This finding was reinforced by the fact that *CERS6* expression is dramatically increased in obese individuals (20). Brüning et al. went on to generate mice lacking *CerS6* in brown adipose tissue and demonstrated that these mice exhibited resolution in hepatic steatosis, improved glucose tolerance, and enhanced mitochondrial β-oxidation and energy expenditure (20). These studies further highlight the importance of ceramide accumulation in BAT in regulating systemic metabolic homeostasis. Using the alternate approach, Hoch et al. found that mice lacking *CerS5*, another enzyme that produces C_16_-ceramides, presented with reduced weight gain and improved systemic metabolic health, including glucose tolerance and white adipose tissue inflammation after high fat diet feeding (70).

## Ceramide Centric Molecular Mechanisms That Impair Adipocyte Function

The broad spectrum of changes observed in adipocyte function due to ceramide intervention results from a series of cell-autonomous ceramide action. We hypothesize that these mechanisms are part of an evolutionarily-conserved pathway that were originally intended to protect cells from excess accumulation of detergent-like fatty acids during times of fuel surplus. Specifically, these activities would reduce mitochondrial efficiency, decrease availability of glucose-and thus increase reliance on fatty acids-for energy production, and block the release of fatty acids from lipid droplets (Figure 2).

### Ceramides Regulate Adipose Tissue Glucose Uptake and Metabolism

In cultured adipocytes, ceramides inhibit insulin-stimulated glucose uptake by blocking the translocation of GLUT4 (71). Consistent with these initial findings, pharmacological inhibition of ceramide biosynthesis via myriocin, adipose tissue-specific reductions in ceramide accumulation via inhibition of SPT2, and DES1 or overexpression of ASAH1 increases adipose tissue glucose uptake and metabolism (6, 35, 40). This result from ceramide's ability to attenuate activation of Akt/PKB, that is obligate for insulin-stimulated glucose uptake (4). Ceramides regulate Akt/PKB by at least two known mechanisms in numerous cell types: First, ceramides activate atypical protein kinase C (PKCζ) which in turn phosphorylates a key residue in the plekstrin homology domain of Akt/PKB, preventing it from being recruited and activated at the plasma membrane in response to insulin (72–74). The second mechanism involves the dephosphorylation of Akt/PKB by protein phosphatase 2A(PP2A). Ceramides may activate PP2A either directly (75), or indirectly by displacing the PP2A inhibitory protein I2PP2A (76). Inhibition of PP2A, with either inhibitor (e.g., okadaic acid) or by overexpressing the SV40 Small T antigen (which blocks access of PP2A to its substrates), negate the effect of ceramides in Akt/PKB in numerous cell types (Figure 2) (77–80).

Of these two known mechanisms, Hajduch et al. demonstrated that in adipocytes the predominant mechanism via which ceramide inhibit Akt/PKB is exclusively mediated by PKCζ (81) as inhibition of PP2A (with okadaic acid) did not prevent ceramide induced insulin action. Moreover, these authors went on to show that adipocytes tend to favor this mechanism due to the preferential sub-localization of ceramide in caveolin enriched membranes (81).

### Ceramides Regulate Adipose Tissue Thermogenic Program

The emerging studies, as discussed above, have highlighted the role of adipose ceramide in the modulation of energy homeostasis. We recently found that ceramide actions on energy homeostasis were due to its ability to selectively impair non-shivering thermogenesis by modulating browning/beiging of the adipose tissue (35). Mechanistically, ceramide analogs were shown to attenuate the expression of key thermogenic genes (e.g., *Ucp1, Pgc1a, Prdm16*, etc.) in beige adipocytes *ex vivo* (35, 55). Conversely, pharmacological intervention that reduced ceramide synthesis in beige adipocytes *ex vivo* increased expression of various thermogenic genes in both rodents and humans. This effect seems to be mediated, at least in part, by ceramides' ability to inhibit lipolysis by blocking the activation of HSL via PP2A (6) (Figure 2).

### Ceramides Regulate Adipose Tissue Mitochondrial Bioenergetics

Ceramides impair mitochondrial function and respiratory capacity by inhibiting oxidative phosphorylation and promoting mitochondrial fragmentation in numerous cell-types including adipocytes (19, 20, 82) (Figure 2). In adipocytes, short-chain ceramide analogs acutely disrupt components of the electron transport chain and β-oxidation (20, 35). Inhibition of genes encoding for SPTLC2, CERS6, and DEGS1 in adipose tissue, results in reductions in adipose tissue ceramide and leads to improvements in mitochondrial energetics (6, 20, 35).

## Outlook and Future Direction

The data described in this review and others in this series, identify ceramides as critical lipid metabolite that modulate adipose tissue function, homeostasis, and contribute to metabolic disease. Moreover, interventions that reduce ceramide synthesis in adipose tissue, delay, or prevent various comorbidities of obesity, such as insulin resistance and liver steatosis. These discoveries, while exciting, raise several essential questions to be answered to validate ceramide as a potential therapeutic target.

First, can additional mechanisms be identified to explain ceramide action? Although initial studies identified a couple of key mechanisms (i.e., regulation of Akt) for ceramide actions, the numerous effects (e.g., regulation of thermogenic program, mitochondrial function) elicited by ceramide seems unlikely to be fully explained solely by PP2A-Akt axis or PKCζ-Akt axis. Therefore, identifying additional molecular mechanisms will be crucial for understanding the roles of ceramides such that the therapeutic strategy could be developed accordingly.

Second, what is the role of immune-cell ceramide content in maintaining adipose tissue homeostasis? How do ceramides regulate these cell populations and how do they interact with adipocytes to gauge nutrient content?

Third, how do β-adrenergic's inhibit ceramide synthesis in adipose tissue? Delineation of the molecular targets downstream of the β-adrenergic receptor may offer additional interventional strategies to target ceramide synthesis.

Fourth, are there additional regulators (nutrients/enzymes) that modulate intracellular ceramide content? The recent development of the ceramide flux assays coupled with secondary biochemical assays might favor the identification of more regulators and lead to additional strategies to safely target ceramide synthesis for its therapeutic use.

Fifth, how various enzymes in sphingolipids synthesis and degradation pathways coordinate to maintain lipid homeostasis, particularly in response to various local and systemic stimuli.

The future research elucidating these important queries holds great promise in not only understanding how ceramides modulate nutrient sensing that underlies metabolic disease processes but also potentially identifying new therapeutic targets to combat metabolic diseases epidemic.

## Author Contributions

YL and BC concieved and wrote the manuscript with inputs from CT. All authors approved it for publication.

## Conflict of Interest

The authors declare that the research was conducted in the absence of any commercial or financial relationships that could be construed as a potential conflict of interest.
